# Metastasiertes Plattenepithelkarzinom auf einem Ulkus bei Graft-versus-Host-Disease nach allogener Stammzelltransplantation

**DOI:** 10.1007/s00105-021-04932-z

**Published:** 2022-01-05

**Authors:** S. Hobelsberger, F. Meier, S. Beissert, S. Abraham

**Affiliations:** Klinik und Poliklinik für Dermatologie, Universitätsklinikum Carl Gustav Carus, Technische Universität Dresden, Fetscherstr. 74, 01307 Dresden, Deutschland

**Keywords:** Marjolin-Ulcus, Immunsuppression, Metastasiertes Plattenepithelkarzinom, Knochenmarktransplantation, Chronische myeloische Leukämie, Marjolin ulcer, Immunosuppression, Metastatic squamous cell carcinoma, Bone marrow transplantation, Chronic myeloid leukemia

## Abstract

Wir berichten über einen 48-jährigen multimorbiden Patienten, der vor 26 Jahren eine allogene Knochenmarktransplantation aufgrund einer chronischen myeloischen Leukämie erhielt; 24 Jahre lang litt der Patient an einer sklerodermiformen chronischen Graft-versus-Host-Disease (GVHD) der Haut und der Lunge mit partieller Lungenresektion und immunsuppressiver Therapie. An den Unterschenkeln entwickelten sich rezidivierende Ulzerationen an den von der kutanen GVHD betroffenen Stellen. Der Patient stellte sich mit einem größenprogredienten Ulkus mit Therapieresistenz in unserer Klinik vor. Histologisch konnte ein Plattenepithelkarzinom diagnostiziert werden. Die Magnetresonanztomographie zeigte eine Knochenbeteiligung und eine kutane In-Transit-Metastase, und die Computertomographie ergab eine Metastase im Os sacrum. Bevor die Therapie eingeleitet wurde, verstarb der Patient plötzlich an den Folgen seiner Vorerkrankungen. Die Entwicklung einer kutanen GVHD ist häufig bei Patienten mit allogener Stammzelltransplantation. Hierbei ist das Risiko für die Entwicklung von Plattenepithelkarzinomen erhöht. Patienten sollten unter engmaschiger dermatologischer Kontrolle stehen. Bei Verdacht auf ein Plattenepithelkarzinom bei vorbestehender GVHD sollte zeitnah eine bioptische Sicherung erfolgen, um das Risiko einer Metastasierung zu senken.

## Abstract (en)

We report the case of a 48-year-old multimorbid man who received allogeneic bone marrow transplantation 26 years ago for chronic myeloid leukemia. For 24 years, the patient suffered from sclerodermiform chronic graft-versus-host disease (GVHD) of the skin and lung with partial lung resection and immunosuppressive therapy. Recurrent ulcerations developed on the lower legs at the sites affected by cutaneous GVHD. The patient presented to our clinic with an ulcer progressive in size with resistance to previous therapy. Histologically, squamous cell carcinoma was found. Magnetic resonance imaging showed bone involvement and cutaneous in-transit metastasis, and computed tomography revealed a metastasis in the sacrum. Before therapy was initiated, the patient died suddenly as a result of his previous illnesses. The development of cutaneous GVHD is common in patients with allogeneic stem cell transplantation. The risk for the development of squamous cell carcinoma is increased. Patients should be under close dermatologic surveillance. If squamous cell carcinoma is suspected in pre-existing GVHD, biopsy should be performed promptly to reduce the risk of metastasis.

## Anamnese

Wir berichten über einen 48-jährigen Patienten mit Zustand nach allogener Knochenmarktransplantation vor 26 Jahren bei chronisch myeloischer Leukämie. Seit 24 Jahren litt der Patient an einer sklerodermiformen chronischen Graft-versus-Host-Disease (GVHD) der Haut mit Sicca-Symptomatik und an einer GVHD der Lunge mit Lungenteilresektion. Fünf Jahre nach Transplantation war aufgrund der progredienten GVHD eine Systemtherapie mit Ciclosporin 200 mg und Mycophenolat-Mofetil 1 g täglich über 1 Jahr verabreicht worden, woraufhin der Befund regredient war. Seit ca. 7 Jahren war die kutane GVHD erneut progredient, besserte sich aber intermittierend unter intensivierter kortikosteroidhaltiger Lokaltherapie und Creme-PUVA (Psoralen plus UV‑A). Die aktuelle Therapie erfolgt seit etwa 4 Jahren mit Prednisolon 5 mg/Tag p.o.

Der Patient berichtete von seit Jahren rezidivierend auftretenden entzündlichen Veränderungen mit Ulzerationen an den Unterschenkeln. Vor 4 Jahren war eine dezente Leitveneninsuffizienz an den Beinen beidseits diagnostiziert worden; eine periphere arterielle Verschlusskrankheit konnte ausgeschlossen werden. Seit 6 Monaten bestand eine Ulzeration am Malleolus medialis rechts mit deutlicher Größenprogredienz. Der Patient führte die Wundversorgung, unterstützt von einer Wundschwester, selbst durch. Zuletzt wurde ein Salbentüll als Wundauflage verwendet. Eine Kompressionstherapie wurde aufgrund zunehmenden Druckschmerzes (8/10 auf der visuellen Analogskala) seit 1 Jahr nicht mehr durchgeführt.

## Klinischer Befund

**Figure 1:**
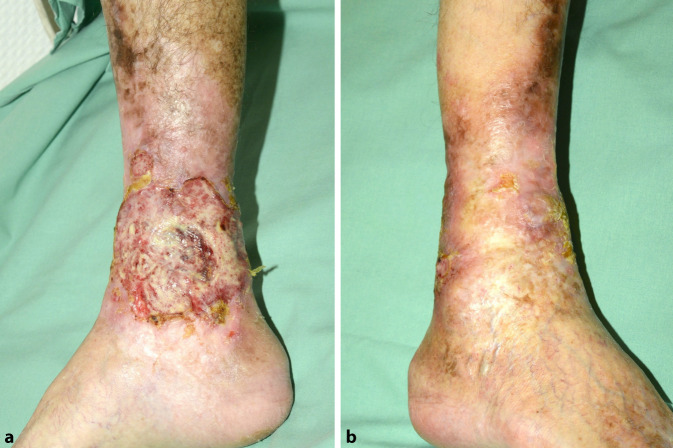

**Figure 2:**
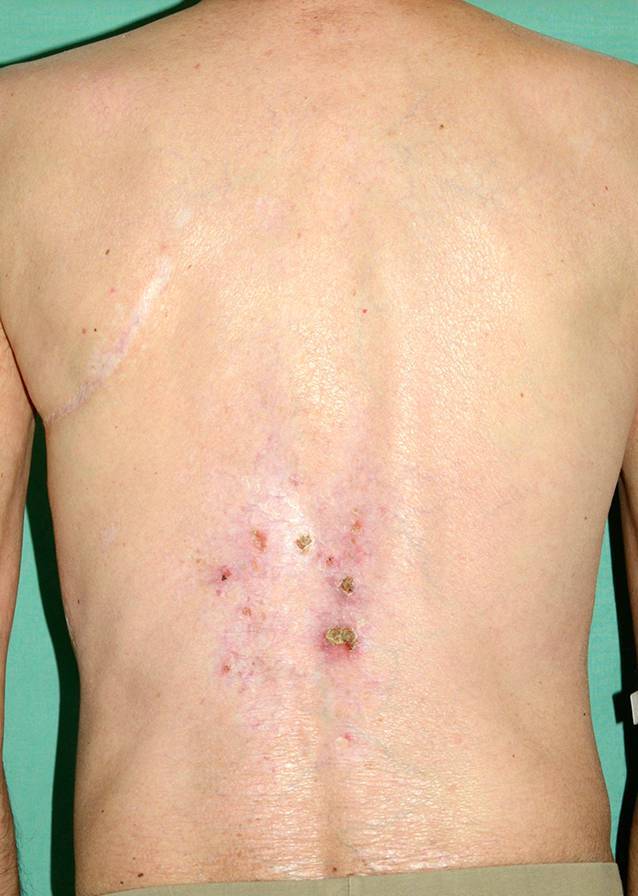

Es zeigte sich am Unterschenkel links medial eine ca. 12 × 8 cm große Ulzeration vom linken Malleolus medialis bis zur dorsalen Ferse reichend sowie am Malleolus lateralis rechts eine 3,2 × 2,5 cm große Ulzeration mit deutlichem Hypergranulationsgewebe (Abb. 1). An den Unterschenkeln beidseits fand sich eine ausgeprägte Induration der Haut mit Narbenbildung und postinflammatorischer Hyperpigmentierung. Auch am mittleren Rücken und pektoral rechts stellte sich die kutane GVHD seit etwa 7 Jahren als erythematöse Plaques mit Hyperkeratosen dar (Abb. 2). Nebenbefundlich fanden sich weißliche Beläge im Bereich der Mundschleimhaut des Rachens im Sinne eines Mundsoors.

## Diagnose

Bei klinischem Verdacht auf ein Plattenepithelkarzinom erfolgten 2 Shavebiopsien der Ulzeration am Unterschenkel rechts.

**Figure 3:**
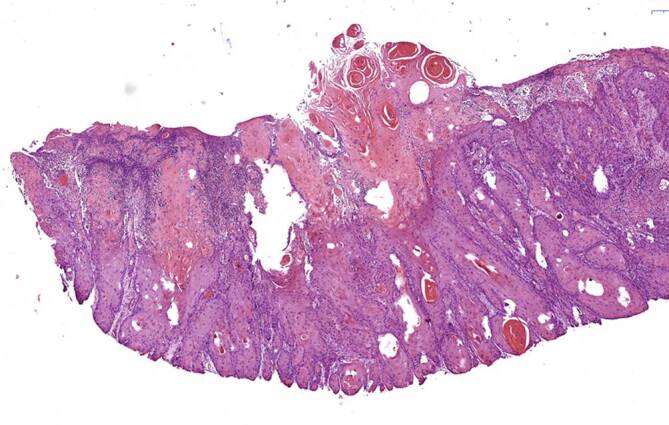

Histologisch zeigte sich ein mittelhoch differenziertes verhornendes Plattenepithelkarzinom mit einer maximalen Tumordicke von 2,3 mm, an dem oberflächlichen Shavebioptat war eine Bestimmung des Invasionslevels nicht möglich, im Oberflächenepithel wurde eine pseudoepitheliomatöse Hyperplasie nachgewiesen (Abb. 3).

**Figure 4:**
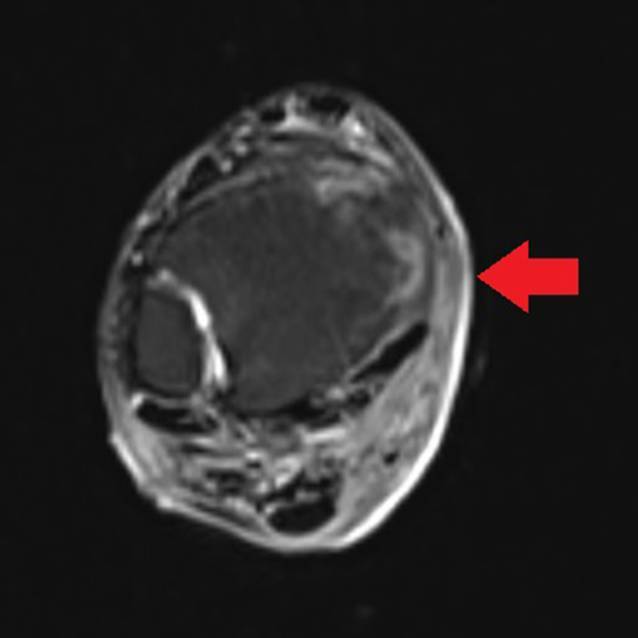

**Figure 5:**
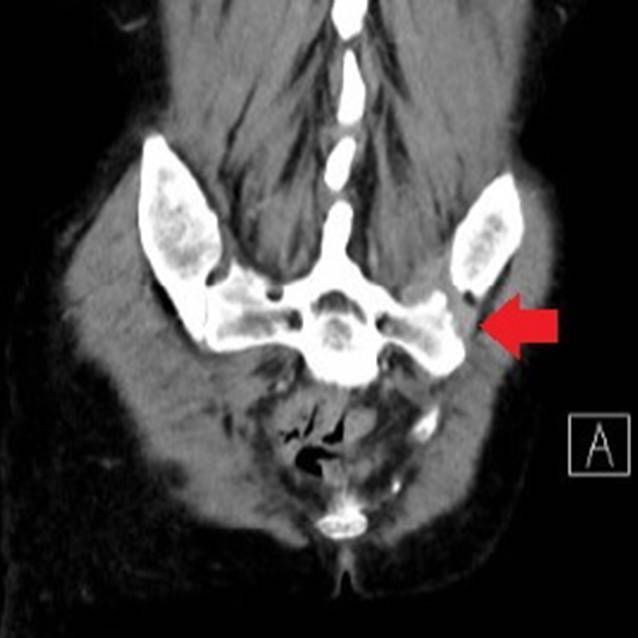

Das MRT des rechten Unterschenkels zeigte eine Knocheninfiltration der Epi- und Metaphyse sowie eine In-Transit-Metastase des rechten Unterschenkels dorsal (Abb. 4). Im Ganzkörper-CT fiel eine metastasenverdächtige Osteolyse im Os sacrum links mit Kontakt zur Iliosakralfuge auf (Abb. 5).

## Therapie und Verlauf

CT-gesteuert wurde ein Knochenzylinder aus dem Os sacrum links entnommen. Die histopathologische Untersuchung ergab eine ausgedehnte Infiltration durch ein mäßig- bis geringgradig differenziertes teils verhornendes Plattenepithelkarzinom, passend zur klinisch vermuteten Metastase. Der Fall wurde in unserer interdisziplinären Tumorkonferenz vorgestellt. Als Therapieoptionen wurden die Amputation bei lokal weit fortgeschrittenem Plattenepithelkarzinom, eine Therapie mit Cemiplimab neoadjuvant unter Berücksichtigung der GVHD sowie die Möglichkeit einer lokal-ablativen Strahlentherapie der Knochenmetastase des Os sacrum besprochen. Der Patient verstarb vor Einleitung einer Therapie, eine Autopsie wurde nicht durchgeführt.

## Diskussion

Patienten mit allogener Stammzelltransplantation entwickeln zu 50 % eine chronische GVHD, meist beginnend im Bereich der Haut [1–3].

Eine chronische GVHD entsteht meistens erst 100 Tage und nicht selten auch noch zwischen 5 und 8 Jahren nach der Transplantation [4, 5].

Sie ist definiert als Syndrom, in dem immunkompetente Spenderzellen die Zellen des transplantierten Patienten „angreifen“, sodass in aller Regel eine immunsuppressive Therapie des Patienten notwendig ist [3]. Es ist die häufigste Komplikation nach einer Transplantation [3].

Eine sklerodermiforme GVHD ist als Stadium 3 der kutanen GVHD nach dem Scoring-System der NIH-Arbeitsgruppe mit einer schlechten Prognose in Bezug auf Sekundärtumoren assoziiert [2, 3, 5].

Die GVHD kann sich auch im Bereich der Mundschleimhaut sowie der Augen- und Genitalschleimhaut, der Leber, des Gastrointestinaltrakts, der Gelenke und Faszien sowie der Lunge manifestieren [2, 5]. Die Konsequenz sind Langzeitkomplikationen mit erhöhter Morbidität und Mortalität [2, 3, 5].

Darüber hinaus haben Patienten mit Stammzelltransplantation ein erhöhtes Risiko für Plattenepithelkarzinome, Basalzellkarzinome und Melanome [1, 6]. DePry et al. identifizierten die Primärdiagnose einer Leukämie oder schweren aplastischen Anämie, jüngeres Alter während der Transplantation, chronische GVHD, verlängerte Immunsuppression und Einsatz von Azathioprin als Risikofaktoren für die Entwicklung von Plattenepithelkarzinomen [1]. Diese betreffen insbesondere die Haut und Mundhöhle [4]. In unserem Fall waren die chronische GVHD mit Bildung eines Ulkus sowie die verlängerte Immunsuppression Risikofaktoren für das entstandene Plattenepithelkarzinom.

Sekundäre Hauttumoren entstehen häufig in den von chronischer kutaner GVHD betroffenen Hautarealen [1, 2].

Die kumulative Inzidenz von kutanen Plattenepithelkarzinomen bei Patienten mit GVHD 20 Jahre nach Transplantation liegt bei 1,1 % [1, 7]. Das mediane Überleben nach einem kutanen Plattenepithelkarzinom beträgt im Durchschnitt 4,1 Jahre, wobei das Plattenepithelkarzinom für etwa ein Drittel der Patienten die primäre oder sekundäre Todesursache ist [1, 7].

Die Pathophysiologie ist noch unklar, es ist aber anzunehmen, dass die chronische Entzündung eine Rolle spielt [1, 2].

Ijaz et al. evaluierten die bestehenden Daten über den Einsatz von Immuntherapien bei Patienten mit allogener Stammzelltransplantation und fanden heraus, dass der Einsatz von Cemiplimab sowohl vor als auch nach allogener Stammzelltransplantation hochwirksam sein kann, die Exposition jedoch zu einem signifikant erhöhten Risiko für GVHD-bedingte Morbidität und Mortalität in dieser Patientenpopulation führen kann [8].

Das im Verlauf entstandene Plattenepithelkarzinom war über einen längeren Zeitraum als Ulcus cruris behandelt worden. Bei therapieresistentem Ulcus cruris, insbesondere bei Vorbestehen einer GVHD mit Immunsuppression, sollte stets an ein Plattenepithelkarzinom gedacht werden. Weiterhin sollten bei einer chronischen GVHD bei erhöhtem Risiko der Entwicklung von nichtmelanozytären Tumoren engmaschige Kontrollen erfolgen (in den ersten 5 Jahren nach Transplantation jährlich, nach Auftreten des ersten Tumors halbjährlich bzw. vierteljährlich) [3].

## Fazit für die Praxis

Die Entwicklung einer kutanen GVHD ist bei Patienten mit allogener Stammzelltransplantation häufig. Aufgrund der GVHD und der meist zusätzlich bestehenden Immunsuppression ist das Risiko für die Entwicklung von Plattenepithelkarzinomen erhöht. Diese Patienten sollten eine engmaschige dermatologische Nachsorge erhalten. Bei Verdacht auf ein Plattenepithelkarzinom bei vorbestehender GVHD ist eine zeitnahe bioptische Sicherung indiziert, um einen lokalen Tumorprogress und eine Metastasierung zu vermeiden. Biopsien aus mehreren Lokalisationen sind empfehlenswert, um das Risiko eines falsch negativen histologischen Befundes zu senken.
